# Estimating the Clinical, Quality-of-Life and Economic Impact of Optimized Management of Type 2 Diabetes Patients in Spain

**DOI:** 10.3390/jcm15041628

**Published:** 2026-02-20

**Authors:** Óscar Martínez-Pérez, Seila Lorenzo-Herrero, Ester Amado-Guirado, Fernando Gómez-Peralta, Jesús Balea-Filgueiras, Joan Barrot, Alberto Cordero, Carlos Crespo, Virginia Pascual, Mónica Cerezales

**Affiliations:** 1 Axentiva Solutions S.L.,08036 Barcelona, Spain; omartinez@axentiva.com; 2 Department of Applied Economics and Quantitative Methods, University of La Laguna, 38200 San Cristóbal de La Laguna, Spain; 3 Axentiva Solutions S.L., 33005 Oviedo, Spain; slorenzo@axentiva.com; 4 Àmbit d’Atenció Barcelona Ciutat, Institut Català de la Salut, 08007 Barcelona, Spain; 5 Endocrinology and Nutrition Unit, Hospital General de Segovia, 40002 Segovia, Spain; 6 Department of Pharmacy, A Coruña University Hospital Complex, 15006 La Coruña, Spain; 7 Primary Health Care Center Dr. Jordi Nadal i Fàbregas (Salt), Gerència d’Atenció Primària, Institut Català de la Salut, 17190 Girona, Spain; 8 Cardiology Department, San Juan University Hospital, 03550 Alicante, Spain; 9 Cardiovascular Research Group (GRINCAVA), Miguel Hernández University, 03202 Elche, Spain; 10 Centre for Biomedical Research Network on Cardiovascular Diseases (CIBERCV), 28029 Madrid, Spain; 11 Department of Genetics, Microbiology and Statistics, University of Barcelona, 08007 Barcelona, Spain; 12 Novo Nordisk Pharma S.A., 28033 Madrid, Spain

**Keywords:** diabetes mellitus, type 2, holistic health, diabetes complications, healthcare costs, quality of life, disability-adjusted life years

## Abstract

**Background**: Type 2 diabetes (T2D) is associated with acute and chronic complications, entailing significant use of healthcare resources. Clinical guidelines recommend holistic management and recognize the critical role of obesity and cardio-renal protection in T2D. This study aims to estimate the clinical, quality of life, and economic benefits of optimized weight, metabolic, and cardiovascular management of T2D-related complications in Spain. **Methods**: An estimation model was built incorporating the risk of complications associated with changes in glycated hemoglobin (HbA1c), weight and high-sensitivity C-reactive protein (hs-CRP), considering incidence of complications and healthcare costs in Spain. A literature review was performed to identify these clinical inputs. The potential reduction in the annual number of complications and their associated disability-adjusted life years (DALYs) and costs were estimated for reductions of 1% HbA1c, 5% weight and 0.5 mg/L hs-CRP in three T2D patient subgroup scenarios. Probabilistic sensitivity analyses were conducted to validate the results and determine their potential range. **Results**: Combined reduction of HbA1c, weight and hs-CRP was estimated to prevent 19.16–20.80% T2D complications per year. This led to an estimated range of 1317–6568 avoided DALYs, and potential annual savings between €242.77M and €821.68M depending on the T2D patient subgroup. Savings per patient and year ranged from €196.86 to €296.75 for the three scenarios analyzed. Sensitivity analysis validated these results. **Conclusions**: Integrated management of patients with T2D, controlling HbA1c levels, weight, and cardiovascular benefit, can improve patient outcomes, reduce incidence of complications, prevent quality of life worsening, and result in cost savings for the Spanish national healthcare system.

## 1. Introduction

Type 2 diabetes (T2D) represents 90% of all diabetes mellitus (DM) types [1,2,3] and is a highly prevalent disease worldwide, even though the rate of underdiagnosis is notable (up to 30%) [1]. Spain has the second highest prevalence in Europe, with around 5 million people affected [1,4]. From 2019, there has been an increase of 42% in this prevalence [1], which might be partially caused by the higher risk of DM associated with Covid-19 infection [5]. This steep slope in DM prevalence is estimated to soften, being around 5% for the 2024–2050 period [6,7].

Current clinical guidelines for T2D recommend a holistic approach focusing on the patient, including glycemic control (reducing glycated hemoglobin (HbA1c)), weight control, and cardio-renal protection as the main treatment objectives [8,9]. Combined therapeutic strategies, including lifestyle modifications and pharmacological treatments, are implemented to achieve these objectives. There is a high prevalence of people living with obesity within the T2D population, and dysfunctional adiposity is considered the leading factor for T2D progression and development of its long-term complications [10]. Weight loss is seen as a critical strategy to improve T2D complications, with 5–15% weight loss established as a primary target [8]. Different studies have shown that poor glycemic control is associated with an increased risk of chronic complications, and intensive glycemic control leads to a decrease in cardiac and microvascular complications in newly diagnosed T2D patients [9,11]. High-sensitivity C-reactive protein (hs-CRP) is an established marker of systemic inflammation associated with increased risk of myocardial infarction or stroke, among other cardiovascular (CV) events, and higher mortality [12,13,14]. In recent years, several antidiabetic agents have demonstrated CV benefit in T2D patients, assessed through hs-CRP and other biomarkers [12,13,15,16]. Considering this evidence, glycemic (HbA1c) and CV control, in addition to weight loss, might lead to a reduction in T2D complications and improvement in survival [17,18].

T2D has been associated with several complications, such as stroke, peripheral vascular disease (PVD), retinopathy, nephropathy, and increased mortality [19]. These complications have a great impact on individuals living with T2D in terms of health-related quality of life (HRQoL), affecting daily life and contributing to early mortality [20]. A common measure of QoL impact of different pathologies is Disability-Adjusted Life Years (DALYs), which account for time lost through premature death and time lived in states of less-than-optimal health. DALYs are a key variable used for evaluating disease burden by the World Health Organization (WHO) [21]. A study assessing the burden of T2D positioned Spain high in the ranking of European countries in terms of DALYs, reporting 1069 DALYs/100,000 inhabitants attributable to T2D, and indicating a significant loss of healthy life years due to the disease and its consequences [22].

T2D complications also entail significant health resource consumption and have been reported to sum up to 37% of the total cost of the disease [2]. For example, eye-related complications conferred an additional yearly cost of €277 per patient, while T2D patients suffering from CV disease required at least twice as many healthcare resources as those without it [23]. Poor glycemic and weight control have also been reported to increase patient management costs [23,24]. In Spain, annual costs for T2D individuals with body mass index (BMI) ≥ 30 kg/m^2^ can increase up to 25% with respect to T2D subjects with lower BMI [10], while annual costs for patients with poor glycemic control (HbA1c ≥8%) increase up to 23% [24]. Therefore, strategies that prevent complications and improve clinical management in people with T2D could improve HRQoL and reduce the associated economic burden, adding a great social value [10,24,25].

Current clinical guidelines include glycemic, weight and comprehensive CV control among the therapeutic objectives of T2D management, but the potential impact of this combined management on T2D complications has not yet been assessed in the Spanish population living with this disease. Thus, our objective was to evaluate the potential effect of integrated management of T2D on disease-related complications. Specifically, this analysis aimed to estimate the potential reductions in T2D complications, as well as the healthcare cost savings and improvement in patient’s HRQoL derived from such reductions as a result of achieving therapeutic objectives for glycemic control, weight loss, and CV benefits in different patient subgroups from the Spanish National Healthcare System (SNHS) perspective.

## 2. Materials and Methods

The objective of this study was to estimate the potential decreases in T2D complications, and their associated improvement in HRQoL and healthcare cost savings, derived from reductions in three clinical parameters: HbA1c, weight and hs-CRP.

### 2.1. Literature Search and Data Extraction

Evidence required for these estimations was retrieved in a retrospective manner through different targeted non-systematic literature searches employing online databases (PubMed, Cochrane Library and Google Scholar) (Table S1 [19,26,27,28,29] and Table S2 [12,14,17,21,23,30,31,32,33,34,35,36,37,38,39,40,41,42,43,44,45,46,47,48,49,50,51,52,53,54,55,56]). The first literature review was performed to identify the most relevant complications related to T2D. The second literature search was launched to find evidence of the association between HbA1c (an established marker of glycemia), weight/BMI, and hs-CRP (a marker of inflammation associated with CV risk [12,13,14]) and the formerly identified complications. Year of publication was not restricted to a specific period to identify all relevant evidence. Literature searches were conducted in 2024 and included articles published in English.

**Figure 1:**
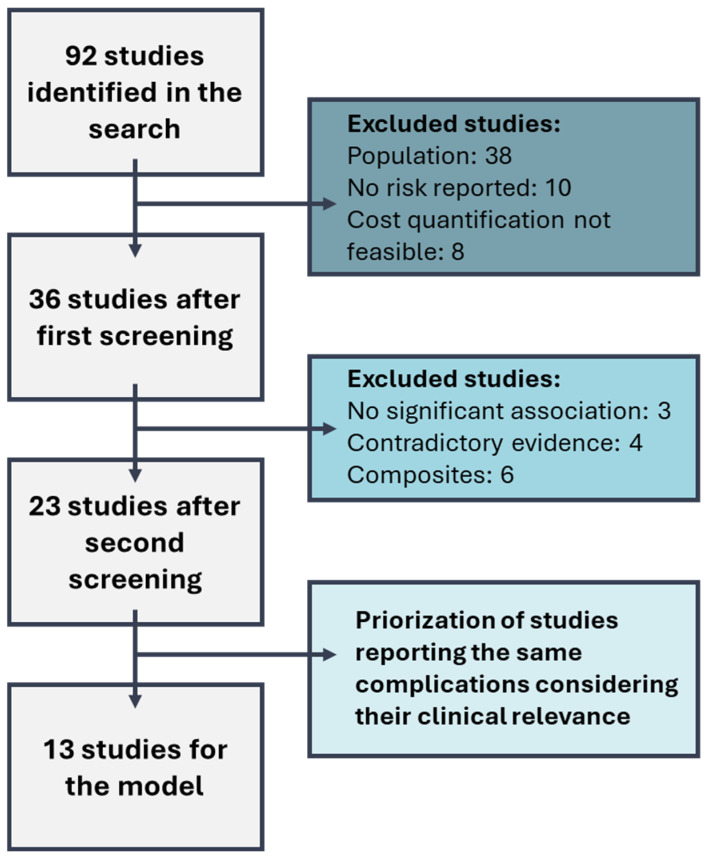
Literature review flow.

Studies were selected based on eligibility criteria, as defined below, and a two-step screening was carried out. The first screening evaluated the studies based on title and abstract, and the second one considered the full text. In the first screening, articles not focusing on T2D population, or not including a specific subgroup analysis for T2D, studies that did not report association of complications with HbA1c levels, weight/BMI or hs-CRP levels, and studies evaluating complications for which cost quantification was not feasible, such as cognitive function, lung function or mental health issues, were excluded (*n* = 56) (Figure 1). In the second screening, studies were discarded (*n* = 13) due to lack of statistically significant evidence of the association between HbA1c, weight/BMI or hs-CRP levels and complications, or because they analyzed composite endpoints instead of individual complications.

The following data was extracted from each study: design, location of study, study population, baseline patient characteristics (such as mean HbA1c, mean BMI or mean hs-CRP), follow-up period, exclusion criteria, analyzed complications, and model characteristics for analysis of the association between HbA1c, weight/BMI or hs-CRP levels and risk of suffering complications, as well as the risk values reported, measured as hazard ratio (HR), odds ratio (OR) or proportion of patients at risk. Some complications were excluded from the model at this step since different (positive, negative, or neutral) associations with HbA1c, weight/BMI and/or hs-CRP were described in different studies and, therefore, there was no conclusive evidence of risk increase/decrease.

Evidence for the association between changes in hs-CRP and CV complications was limited (*n* = 3). A meta-analysis using a fixed-effect inverse-variance method was performed to incorporate all the studies reporting risk of CV complications based on hs-CRP. This fixed-effect approach was chosen given that no heterogeneity was observed between the studies included for the meta-analysis. A fixed-effect meta-analysis provided a more suitable approach that reduced selection bias, since it synthesizes the scarce evidence available.

Additionally, a review to find incidences of each complication in T2D patients in Spain was carried out, as well as a search to determine the interaction between HbA1c, body weight or BMI, and/or hs-CRP in T2D.

DALYs associated with each complication were also identified from the literature (Table S3 [21,54,55]) and when possible, estimates reported for mild cases were selected in order to minimize overestimation.

Costs for each complication within the Spanish National Healthcare System (SNHS) were defined using only diagnosis-related groups (DRGs), as disclosed for 2023 (38th DRG version) and inflated to €2025 (Table S4 [56]). These DRG costs comprise direct medical costs only.

### 2.2. Modeling and Statistical Analysis

The model framework defined for this analysis allows for quantification of the burden of the defined complications associated with T2D, and the impact of integrated patient management on the incidence and costs of these complications.

**Figure 2:**
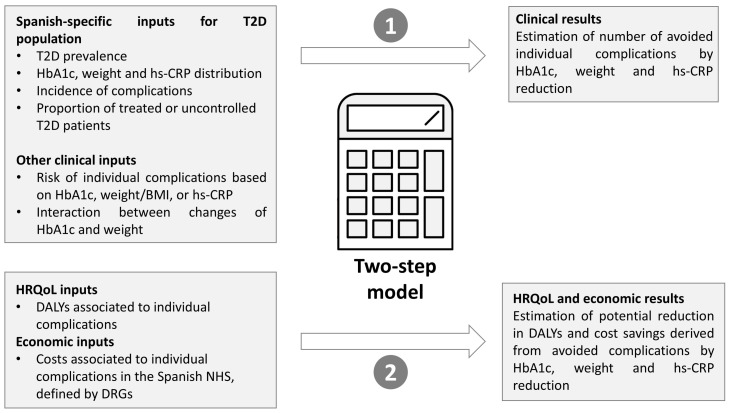
Input and results flow of the estimation model. BMI: body mass index; DALYs: disability-adjusted life years; DRG: diagnosis-related group; HbA1c: glycated hemoglobin; HRQoL: health-related quality of life; hs-CRP: high sensitivity C-reactive protein; NHS: national healthcare system; T2D: type 2 diabetes.

The flow of the model is depicted in Figure 2. In this analysis, 1% HbA1c reduction + 5% weight reduction + 0.5 mg/L hs-CRP reduction was evaluated, as these are common treatment targets for current therapeutic alternatives or endpoints included in clinical trials [57]. Three different patient scenarios were considered to estimate potential benefits: (I) treated Spanish T2D patients [51]; (II) treated Spanish T2D patients with poor glycemic control [52]; (III) treated Spanish T2D patients with BMI ≥ 30 kg/m^2^ and poor glycemic control [53], as a combined therapeutic objective recommended by clinical guidelines. All scenarios were calculated based on the prevalent 2025 Spanish T2D population [4,49] and were compared to their own base case, where no HbA1c, weight, or hs-CRP reduction was applied.

According to risks retrieved from the literature and using the incidence of complications from T2D patients in Spain, the estimated risk reduction of each complication when varying HbA1c, weight/BMI and hs-CPR levels was calculated. Based on the European survey reporting healthcare data in Spain in 2020 (*n* = 1988) [44], the weight and height distribution of the T2D population in Spain was extracted in order to estimate the effect of changes in the real distribution of the population. The distributions of HbA1c and hs-CRP levels among the Spanish population were simulated based on the literature [45,46,47].

With all this data, new incidences of complications when reducing HbA1c, weight and hs-CRP were calculated, considering the interaction between weight and HbA1c found in the literature [48]. Such interaction translated into values of 5% weight reduction associated with 0.44% HbA1c reduction in our population. The corresponding consequences of an additional 0.56% HbA1c reduction (required to reach a total 1% decrease) was subsequently added to the calculations. No interaction with hs-CRP was considered in the model due to the lack of evidence in the literature regarding potential interactions among these three variables. As a conservative approach, incidence reduction was not calculated as a fully additive model. Estimates of weight and HbA1c risk reductions were calculated using a conservative maximin-like decision rule, selecting the highest effect among individuals’ reduction, thus assuming non-additivity to avoid overestimation. Incidence reduction derived from hs-CRP was then added as an additional CV benefit to that already identified from HbA1c and weight control. The model had a one-year time horizon. DALYs avoided and cost savings were also estimated for the patient subgroups for each individual complication and for all the complications avoided as a result of weight loss and HbA1c and hs-CRP reductions. A bottom-up gross-costing methodology was employed for cost-saving estimations incorporating complication costs per DRG.

Different scenarios were evaluated to obtain potential ranges of complications reduced by optimized treatment targets and, consequently, potential reduced costs and improved HRQoL. A probabilistic sensitivity analysis was carried out with 1000 simulations based on the distribution of complication risks to estimate distributions of avoided cases and DALYs, and cost savings. Log-normal distributions were used for hazard ratios, and beta distributions were applied for probabilities. All statistical analyses were carried out using Microsoft^®^ Excel^®^ for Microsoft 365 MSO (Version 2601 Build 16.0.19628.20166) 64-bit.

No specific checklist is available for the analysis performed in this study; however, given it applies certain elements commonly found in cost-of-illness studies, a consensus checklist for this type of study is included to demonstrate that this analysis is in line with established methodological guidelines (Table S5) [58].

## 3. Results

Evidence was found for the association of changes in HbA1c levels with myocardial infarction, stroke, PVD, chronic kidney disease, retinopathy, and dementia; for the association of changes in body weight/BMI with myocardial infarction, heart failure, stroke, PVD, chronic kidney disease, diabetic neuropathy, dementia, and cancer; and for the association of changes in hs-CRP levels with myocardial infarction and stroke.

**Figure 3:**
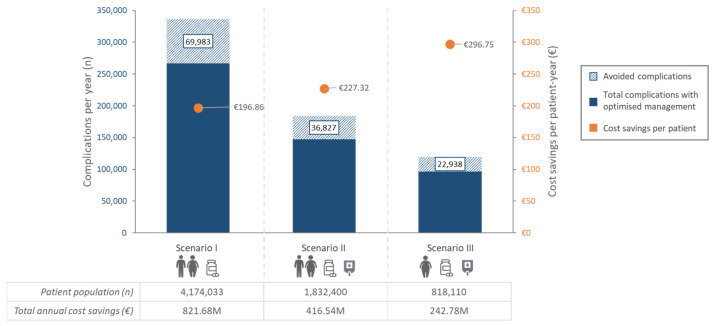
Total number of complications and their corresponding avoided complications and cost savings per patient derived from reductions in HbA1c (1%), weight (5%) and hs-CRP (0.5 mg/L) for each scenario. Scenario I comprised all treated Spanish T2D patients; scenario II comprised treated Spanish T2D patients with poor glycemic control; and scenario III comprised Spanish T2D patients with BMI ≥ 30 kg/m^2^ and poor glycemic control. Estimated avoided complications per scenario correspond to the stripped area. M: millions.

The estimated number of patients for each scenario was as follows: (I) 4,174,033 treated Spanish T2D patients; (II) 1,832,400 treated Spanish T2D patients with poor glycemic control; and (III) 818,110 treated Spanish T2D patients with BMI ≥ 30 kg/m^2^ and poor glycemic control (Figure 3).

Clinical results in terms of reductions in the annual number of individual complications can be seen in Table 1 and Figures S1–S3. In scenario I, there were 69,983 (estimated range of 27,134–107,591) total avoided complications, corresponding to a 20.80% (8.07–31.98%) reduction compared to the base case (Figure 3). Avoided complications in scenario II were estimated to be 36,827 (16,928–52,610), 19.98% (9.18–28.54%) less than in the base case; estimations for scenario III accounted for 22,938 (12,388–31,553) avoided complications, a 19.16% (10.35–26.35%) reduction in the base case (Figure 3).

**Figure 4:**
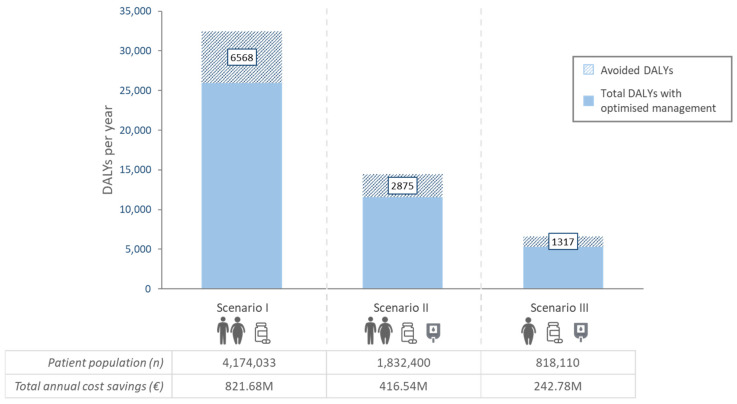
Total number of DALYs and their corresponding avoided DALYs derived from reductions in HbA1c (1%), weight (5%) and hs-CRP (0.5 mg/L) for each scenario. Scenario I comprised all treated Spanish T2D patients; scenario II comprised treated Spanish T2D patients with poor glycemic control; and scenario III comprised Spanish T2D patients with BMI ≥ 30 kg/m^2^ and poor glycemic control. Estimated avoided DALYs per scenario correspond to the stripped area. DALY: disability-adjusted life year; M: millions.

Considering HRQoL, the reduction in complications was estimated to prevent 6568 DALYs (1784–10,524) for scenario I, 2875 DALYs (808–4460) for scenario II, and 1317 DALYs (385–2033) for scenario III per year (Figure 4). This represents a range between 19.85% and 20.21% avoided DALYs compared to the base case and between 1.57 and 1.61 avoided DALYs per 1000 patient-years. Avoided DALYs per individual complication are listed in Table 2.

Regarding the economic impact of avoiding complications and the related potntial cost savings (Table 3), estimations ranged between €242.77 millions (M) (€109.63M–€348.37M) and €821.68M (€243.35M–€1285.31M) avoided costs per year for scenarios III and I, respectively (Figure 3). This corresponds to potential cost savings between 20.67% and 22.92% compared to the base case. Annual savings per patient range between €196.86 and €296.75, with the highest value corresponding to scenario III (Figure 3).

The results obtained from the probabilistic sensitivity analysis (Figure S4) provide more information about the distribution of avoided cases and savings in each scenario. These distributions allowed us to narrow the range of avoided cases and costs, making the estimate more precise. For example, in the case of scenario III, it showed that the majority of simulations fell between 21,062 and 26,003 avoided complications, between 1120 and 1651 avoided DALYs, and between €215.99M and €286.30M potential savings (Figure S4).

## 4. Discussion

Previous studies have assessed the consequences of optimized glycemic control in T2D patients, unveiling benefits in terms of reduction in complications and healthcare costs [59,60,61]. However, clinical guidelines recommend an integrated patient-centered approach, taking into account not only glycemia, but other therapeutic targets such as weight and CV risk in T2D management [8,9]. This is the first study that brings to light the potential clinical, economic and HRQoL-related advantages of the combined control of HbA1c, weight and hs-CRP on the incidence, DALYs and costs of complications in people living with T2D.

According to our estimations, reductions of 1% HbA1c, 5% weight and 0.5 mg/L hs-CRP could prevent around 20% complications in three T2D patient subgroups in Spain. In all the analyzed scenarios, incidence of PVD, followed by stroke and myocardial infarction, showed large reductions. In line with these results, overweight and obesity are widely considered relevant risk factors for macrovascular complications in T2D patients [62]. Likewise, a recent systematic review described a reduced risk of major cardiac adverse events and stroke linked to treatment with antidiabetic drugs previously reported to improve glycemic and weight control and with demonstrated CV benefits [63]. Indeed, the last update of the List of Essential Medicines published by the WHO included glucagon-like peptide-1 (GLP-1) receptor agonists as an add-on therapy in T2D, acknowledging the healthcare challenge posed by obesity, and the increased risk of CV disease, in people living with DM [64]. Based on our estimations and given that the prevalence of T2D-related complications, including CV disease, is steadily increasing in Spain [51], a management strategy comprising weight, HbA1c, and hs-CRP control might prove valuable for the SNHS.

T2D has a detrimental effect on HRQoL, severely worsened by its associated complications [65]. Hence, the reduction in complications derived from optimized management comprising HbA1c, weight and hs-CRP control could improve HRQoL in T2D patients, estimated herein at around 1.6 DALYs avoided per 1000 patient-years across all scenarios. This translates into more time lived with better health status compared to patients suffering from complications. Treatment with distinct antidiabetic drugs with demonstrated clinical benefits for glycemic, weight and CV control already demonstrated HRQoL improvement, reinforcing the relevance of integrated T2D management [66].

In this study, a major proportion of the total avoided DALYs came from reducing the cases of diabetic neuropathy and chronic kidney disease, chronic complications linked to a substantial deterioration in HRQoL [67,68,69]. It is important to note that chronic complications affect T2D patients over the years and short-term complications might imply sequelae in the long term, in addition to their impact on mortality risk. For instance, patients suffering from PVD can develop chronic ischemia resulting in limb amputation, while severe retinopathy can cause blindness [70,71,72,73]. Furthermore, DALYs do not account for other aspects related to quality of life, such as the impact of complications on the daily life and emotional wellbeing of the patient. Consequently, avoiding T2D complications is likely to positively impact HRQoL in other dimensions and beyond the time horizon of one year analyzed in this study.

In economic terms, Mata-Cases et al. already demonstrated that optimized glycemic control could lead to cost savings for the SNHS because of a reduction in complications in T2D patients [59]. Our results further unveil the potential benefits of integrated management considering not only glycemia, but also weight and CV risk. The avoided complications derived from this management translated into potential annual cost savings of up to €821M for the SNHS. These savings are mainly driven by reduced costs of severe complications associated with large healthcare expenditures, namely diabetic neuropathy and retinopathy. It is important to note that the time horizon considered in the analysis limits the potential savings to one year, and reflect only the management for acute events of these complications, identified by DRGs, representing a conservative perspective. Thus, longer-term cost savings associated with chronic complications—which could comprise savings not only for hospital care but also for outpatient and primary care management, as well as treatments—are not accounted for in these estimations. In this context, a previous study in Saudi Arabia estimated savings of around $23,000/patient for each 1% HbA1c reduction over 10 years due to prevention of T2D-related complications [60], suggesting that integrated patient management could represent higher savings for the SNHS over a longer term.

Around 44% of patients living with T2D in Spain are not reaching recommended glycemic goals, and more than 50% exhibit a BMI higher than 30 kg/m^2^ [51,52]. Healthcare costs are higher for these patients, who also exhibit a greater frequency of complications [18,46]. Spanish T2D patients also exhibit a two-fold increased risk of CV complications, with a corresponding increase in healthcare costs [23]. Combined reductions of 1% HbA1c, 5% weight and 0.5 mg/L hs-CRP could save 20.67% of costs related to complications in uncontrolled patients with obesity according to our model, corresponding to higher estimated savings per patient compared with the other subgroups. Previously published studies outlined the burden of T2D complications in Spain, and their costs represented around 37% of the total costs of T2D, most of them corresponding to CV complications [2,23,51,74,75], and highlighting the need for patient-centered management that can limit excessive use of healthcare resources.

Importantly, the estimated cost savings presented in this study consider the impact of glycemic, weight, and CV control on complications. These savings could be superior for the SNHS given that there are other cost drivers in patients living with T2D. Indeed, an observational study at a national level reported economic benefits linked to BMI reduction thanks to lower pharmacy costs [76]. In addition, T2D complications have a social impact, leading to indirect costs derived from work absenteeism [77,78], which could be diminished by integrated patient management. Further research is needed to evaluate the societal advantages of such an approach.

### Limitations

This study provides a modeling approach to estimate the impact of integrated T2D management due to the lack of contemporary prospective real-world studies addressing this question in Spain. Consequently, it has certain limitations derived from the available literature used to build the model. It is important to note that the studies reporting inputs incorporated in the model have their own limitations, which will not be addressed here. Given the limited evidence available for hs-CRP, a meta-analysis was conducted to avoid selection bias, since a single study might not be robust enough to account for its effect on T2D complications. It is important to note that this methodology has limitations due to the additional modeling process, but a conservative approach was applied throughout the study to minimize overestimations.

Risks of each complication based on HbA1c, weight or hs-CRP levels were incorporated as independent variables in the estimation model. No evidence reporting the risk of suffering more than one complication at a time was found. The model was built based on the reported interaction between weight and HbA1c in T2D, avoiding the assumption of an additive effect. The impact of hs-CRP was considered additive, since it was assumed that its control provided an extra CV benefit to that was already achieved with glycemic and weight management. Of note, no interaction between the three variables was found.

Additionally, both risk reduction and incidence studies generally report data on complications in the entire population with T2D. Since the scenarios assess subgroups within the total population, there could be an underestimation of the potential effects. Therefore, while this emerges as a limitation, it also presents a more conservative view of the results. For those studies reporting patient subgroup analysis, this limitation is minimized, given that the HbA1c and weight/BMI distributions were properly adjusted to each scenario. It is also worth noting that HbA1c reduction was applied to the total patient group in scenario I, which includes those patients with good glycemic control. Reducing glycemia below certain levels can be detrimental to the patient, affecting their health condition and potentially translating into increased mortality rates and healthcare costs. This limitation did not affect scenarios II and III since the analysis was restricted to poorly controlled patients.

No specific DRGs for diabetic neuropathy and retinopathy are defined. Thus, costs were approximated as the highest level of severity (level 4) within the DRG closest to the complication (i.e., infections and other ocular diseases for retinopathy), which can be overestimated in the model. Yet, an early study evaluating the economic burden of T2D complications estimated similar costs for neuropathy and superior costs for retinopathy [2], supporting the approach applied in our model.

Finally, it is important to acknowledge that the potential cost savings estimated in this analysis were directly derived from avoided complications in T2D patient subgroups, without considering any costs the SNHS would incur associated with patient management and treatment to achieve the evaluated therapeutic goals. Also, these complication costs were implemented in the model from the beginning of the one-year time horizon, although events might be avoided at different time points in a real-world setting, which would limit the associated savings. Probabilistic sensitivity analyses were conducted to provide a lower range for the economic benefits described to partially account for these.

Despite these limitations, a similar structure to that of cost-of-illness analyses was employed in this study to ensure understanding and transparency of the results, following a previous consensus guideline defining key aspects of this type of analysis (Table S5) [36]. Still, research focusing on the burden and costs of T2D and its current management from a Spanish perspective is needed in order to update the available evidence and provide a basis for healthcare decisions and policies regarding this patient population in Spain.

## 5. Conclusions

This study highlights the significant advantages of integrated T2D management at the clinical, HRQoL and economic levels, emphasizing the combined control of HbA1c, weight and hs-CRP. Achieving a combined reduction of 1% HbA1c, 5% weight and 0.5 mg/L could potentially reduce complications by nearly 21% in Spanish T2D patients. Economically, this strategy could save over €800 M annually by lowering the incidence of severe complications like diabetic neuropathy and retinopathy. Despite limitations, our findings strongly support the need for integrated T2D care strategies that could significantly improve patient health outcomes and lead to important cost savings for the SNHS. Future research should further explore the benefits of wider comprehensive management, including renal control, to provide a more comprehensive understanding of its impact. Implementing these strategies could enhance the quality of life for T2D patients and ease the economic burden on the healthcare system.

## Figures and Tables

**Table 1:** Estimated total and avoided number of individual complications for the three scenarios analyzed.

	Scenario I				Scenario II				Scenario III			
Complication	Base Case	Optimized Management	Complications Avoided	Reduction (%)	Base Case	Optimized Management	Complications Avoided	Reduction (%)	Base Case	Optimized Management	Complications Avoided	Reduction (%)
Myocardial infarction, *n* [estimated range]	1505	1099 [938–1307]	405 [198–567]	26.94% [13.17–37.67%]	661	483 [412–574]	178 [87–249]	26.94% [13.17–37.67%]	295	216 [184–256]	79 [39–111]	26.89% [13.12–37.63%]
Heart failure, *n* [estimated range]	3731	3642 [3576–3694]	89 [37–154]	2.39% [0.99–4.13%]	1638	1599 [1570–1622]	39 [16–68]	2.39% [0.99–4.13%]	731	679 [648–708]	52 [23–83]	7.12% [3.17–11.33%]
Stroke, *n* [estimated range]	2097	1423 [1165–1713]	675 [384–932]	32.17% [18.31–44.46%]	921	624 [511–752]	296 [169–409]	32.17% [18.31–44.46%]	411	279 [228–336]	132 [75–183]	32.17% [18.31–44.46%]
Peripheral vascular disease, *n* [estimated range]	15,778	9083 [6938–11,919]	6695 [3859–8840]	42.43% [24.46–56.03%]	6926	3987 [3046–5232]	2939 [1694–3881]	42.43% [24.46–56.03%]	3092	1780 [1360–2336]	1312 [756–1733]	42.43% [24.46–56.03%]
Chronic kidney disease, *n* [estimated range]	49,568	41,526 [39,313–43,849]	8042 [5719–10,255]	16.22% [11.54–20.69%]	21,760	18,230 [17,258–19,250]	3531 [2511–4502]	16.22% [11.54–20.69%]	9715	8139 [7705–8594]	1576 [1121–2010]	16.22% [11.54–20.69%]
Diabetic neuropathy, *n* [estimated range]	159,654	119,303 [94,747–151,905]	40,351 [7749–64,907]	25.27% [4.85–40.65%]	70,088	52,374 [41,594–66,686]	17,714 [3402–28,494]	25.27% [4.85–40.65%]	31,292	23,383 [18,570–29,773]	7909 [1519–12,722]	25.27% [4.85–40.65%]
Dementia, *n* [estimated range]	28,453	27,073 [21,811–28,453]	1380 [0–6642]	4.85% [0–23.34%]	12,491	12,491 [12,491–12,491]	0 [0–0]	0% [0–0%]	5577	5577 [5577–5577]	0 [0–0]	0% [0–0%]
Retinopathy, *n* [estimated range]	64,178	52,077 [49,244–55,115]	12,101 [9063–14,934]	18.86% [14.12–23.27%]	64,831	52,809 [49,982–55,837]	12,022 [8995–14,850]	18.54% [13.87–22.91%]	66,377	54,548 [51,736–57,547]	11,829 [8831–14,642]	17.82% [13.3–22.06%]
Cancer, *n* [estimated range]	11,446	11,201 [11,087–11,321]	245 [126–359]	2.14% [1.1–3.14%]	5025	4917 [4867–4970]	108 [55–158]	2.14% [1.1–3.14%]	2243	2195 [2173–2219]	48 [25–70]	2.14% [1.10–3.14%]

**Table 2:** Estimated total and avoided DALYs per complication in the three scenarios analyzed.

	Scenario I				Scenario II				Scenario III			
Complication	Base Case	Optimized Management	DALYs Avoided	Reduction (%)	Base Case	Optimized Management	DALYs Avoided	Reduction (%)	Base Case	Optimized Management	DALYs Avoided	Reduction (%)
Myocardial infarction, DALYs [estimated range]	114	84 [71–99]	31 [15–43]	26.94% [13.16–37.67]	50	37 [31–44]	14 [7–19]	26.94% [13.16–37.67%]	22	16 [14–19]	6 [3–8]	26.94% [13.16–37.67]
Heart failure, DALYs [estimated range]	153	149 [147–151]	4 [2–6]	2.39% [0.99–4.13]	67	66 [64–66]	1.6 [1–3]	2.39% [0.99–4.13%]	30	28 [27–29]	2 [1–3]	7.12% [3.17–11.33]
Stroke, DALYs [estimated range]	40	27 [22–33]	13 [7–18]	32.17% [18.31–44.46]	17	12 [10–14]	6 [3–8]	32.17% [18.31–44.46%]	8	5 [4–6]	3 [1–3]	32.17% [18.31–44.46]
Peripheral vascular disease, DALYs [estimated range]	214	123 [94–162]	91 [52–120]	42.43% [24.46–56.03]	94	54 [41–71]	40 [23–53]	42.43% [24.46–56.03%]	42	24 [18–32]	18 [10–24]	42.43% [24.46–56.03]
Chronic kidney disease, DALYs [estimated range]	5159	4322 [4091–4563]	838 [596–1068]	16.22% [11.54–20.69%]	2265	1897 [1796–2004]	367 [261–469]	16.22% [11.54–20.69%]	1011	847 [802–894]	164 [117–209]	16.23% [11.55–20.70]
Diabetic neuropathy, DALYs [estimated range]	21,234	15,867 [12,601–20,203]	5367 [1031–8633]	25.27% [4.85–40.65%]	9322	6966 [5532–8869]	2356 [452–3790]	25.27% [4.85–40.65%]	4162	3110 [2470–3960]	1052 [202–1692]	25.27% [4.85–40.65%]
Dementia, DALYs [estimated range]	1963	1868 [1506–1963]	95 [0–458]	4.85% [0–23.34%]	862	862 [862–862]	0 [0–0]	0% [0–0%]	385	385 [385–385]	0 [0–0]	0% [0–0%]
Retinopathy, DALYs [estimated range]	321	260 [246–275]	61 [45–75]	18.86% [14.12–23.27%]	324	264 [250–279]	60 [45–74]	18.54% [13.87–22.91%]	332	273 [259–288]	59 [44–73]	17.82% [13.3–22.06%]
Cancer, DALYs [estimated range]	3296	3226 [3193–3260]	71 [36–103]	2.14% [1.1–3.14%]	1447	1416 [1402–1431]	31 [16–45]	2.14% [1.10–3.14%]	646	632 [626–639]	14 [7–20]	2.14% [1.10–3.14%]

**Table 3:** Estimated total and avoided costs per complication in the three scenarios analyzed.

	Scenario I				Scenario II				Scenario III			
Complication	Base Case	Optimized Management	Cost Savings	Reduction (%)	Base Case	Optimized Management	Cost Savings	Reduction (%)	Base Case	Optimized Management	Cost Savings	Reduction (%)
Myocardial infarction, € [estimated range]	6.02M	4.39M [3.75M–5.22M]	1.62M [0.79M–2.27M]	26.94% [13.17–37.67%]	2.64M	1.93M [1.64M–2.29M]	0.71M [0.35M–0.99M]	26.94% [13.17–37.67%]	1.18M	0.86M [0.73M–1.02M]	0.32M [0.15M–0.44M]	26.89% [13.12–37.63%]
Heart failure, € [estimated range]	1.40M	13.70M [13.45M–13.90M]	0.33M [0.14M–0.58M]	2.39% [0.99–4.13%]	6.16M	6.01M [5.90M–6.10M]	0.15M [0.06M–0.25M]	2.39% [0.99–4.13%]	2.75M	2.55M [2.44M–2.66M]	0.19M [0.09M–0.31M]	7.12% [3.17–11.33%]
Stroke, € [estimated range]	18.58M	12.60M [10.32M–15.18M]	5.98M [3.40M–8.26M]	32.17% [18.31–44.46%]	8.16M	5.53M [4.53M–6.66M]	2.62M [1.49M–3.63M]	32.17% [18.31–44.46%]	3.64M	2.47M [2.02M–2.97M]	1.17M [0.67M–1.62M]	32.17% [18.31–44.46%]
Peripheral vascular disease, € [estimated range]	62.99M	36.26M [27.70M–47.58M]	26.73M [15.41M–35.29M]	42.43% [24.46–56.03%]	27.65M	15.92M [12.16M–20.89M]	11.73M [6.76M–15.49M]	42.43% [24.46–56.03%]	12.34M	7.11M [5.43M–9.33M]	5.24M [3.02M–6.92M]	42.43% [24.46–56.03%]
Chronic kidney disease, € [estimated range]	169.94M	142.37M [134.78M–150.34M]	27.57M [19.61M–35.16M]	16.22% [11.54–20.69%]	74.60M	62.50M [59.17M–66.00M]	12.10M [8.61M–15.43M]	16.22% [11.54–20.69%]	33.31M	27.90M [26.42M–29.46M]	5.40M [3.84M–6.89M]	16.22% [11.54–20.69%]
Diabetic neuropathy, € [estimated range]	2552.91M	1907.69M [1515.03M–2429.01M]	645.23M [123.90M–1037.88M]	25.27% [4.85–40.65%]	1120.73M	837.47M [665.10M–1066.33M]	283.25M [54.39M–455.63M]	25.27% [4.85–40.65%]	500.37M	373.91M [296.94M–476.08M]	126.46M [24.28M–203.42M]	25.27% [4.85–40.65%]
Dementia, € [estimated range]	141.89M	135.01M [108.77M–141.89M]	6.88M [0–33.12M]	4.85% [0–23.34%]	62.29M	62.29M [62.29M–62.29M]	0 [0–0]	0% [0–0%]	27.81M	27.81M [27.81M–27.81M]	0 [0–0]	0% [0–0%]
Retinopathy, € [estimated range]	562.86M	456.73M [431.89M–483.38M]	106.13M [79.48M–130.98M]	18.86% [14.12–23.27%]	568.59M	463.16M [438.36M–489.71M]	105.44M [78.89M–130.24M]	18.54% [13.87–22.91%]	582.15M	478.41M [453.74M–504.71M]	103.74M [77.45M–128.41M]	17.82% [13.3–22.06%]
Cancer, € [estimated range]	56.40M	55.19M [54.63M–55.78M]	1.21M [0.62M–1.77M]	2.14% [1.1–3.14%]	24.76M	24.23M [23.98M–24.49M]	0.53M [0.27M–0.78M]	2.14% [1.1–3.14%]	11.05M	10.82M [10.71M–10.93M]	0.24 [0.12M–0.35M]	2.14% [1.1–3.14%]

## Data Availability

The data used in this study is publicly available in the corresponding studies referenced in the article.

## Supplementary Materials

The following supporting information can be downloaded at: https://www.mdpi.com/article/10.3390/jcm15041628/s1. Table S1: Literature search; Table S2: Sources of inputs included in the model; Table S3: DALYs associated with complications; Table S4: Costs associated with complications based on DRGs (€, as of 2025); Table S5: Consensus-based checklist for cost-of-illness studies [37]; Figure S1. Percentage of reduction for each individual complication estimated for scenario I; Figure S2. Percentage of reduction for each individual complication estimated for scenario II; Figure S3. Percentage of reduction for each individual complication estimated for scenario III; Figure S4. Patient distribution of the estimated number of avoided complications and their potential avoided DALYs and cost savings according to probabilistic sensitivity analyses. References [4,12,14,17,19,21,23,26,27,28,29,30,31,32,33,34,35,36,37,38,39,40,41,42,43,44,45,46,48,50,51,52,53,54,55,56,58] are cited in the Supplementary Materials.

## Abbreviations

The following abbreviations are used in this manuscript:

BMI	body mass index
CV	cardiovascular
DALY	disability-adjusted life year
DM	diabetes mellitus
DRG	diagnosis-related group
HbA1c	glycated hemoglobin
HR	hazard ratio
HRQoL	health-related quality of life
hs-CRP	high sensitivity C-reactive protein
OR	odds ratio
PVD	peripheral vascular disease
SNHS	Spanish national healthcare system
T2D	type 2 diabetes
