# Supplementary material for: Estimating the Clinical, Quality-of-Life and Economic Impact of Optimized Management of Type 2 Diabetes Patients in Spain
Source: J Clin Med. 2026 Feb 20;15(4):1628. doi: 10.3390/jcm15041628 (PMC12941770; doi:10.3390/jcm15041628)
Supplement: Supplementary file 1 [file jcm-15-01628-s001.zip › jcm-4129010-supplementary.pdf]

Table S1 *Literature search*

First, the most relevant complications associated with type 2 diabetes (T2D) were identified based on the available literature, as shown in the following table. For this purpose, different online databases were employed (PubMed, Cochrane Library and Google Scholar) to contrast the evidence found.

Complication identified	Source
Myocardial Infarction	Shah *et al*., 2022[1]; Tomic *et al*., 2022[2]
Heart failure	Shah *et al*., 2022[1]; Tomic *et al*., 2022[2]
Stroke	Tomic *et al*., 2022[2]; Shah *et al*., 2022[1]; Zheng *et al*., 2017[3]
Peripheral vascular disease	Lu *et al*., 2023[4]; Tomic *et al*., 2022[2]
Chronic kidney disease	Lu *et al*., 2023[4]; Tomic *et al*., 2022[2]; Zheng *et al*., 2017[3]
Diabetic neuropathy	Lu *et al*., 2023[4]; Tomic *et al*., 2022[2]; Zheng *et al*., 2017[3]
Retinopathy	Lu *et al*., 2023[4]; Tomic *et al*., 2022[2]; Zheng *et al*., 2017[3]
Dementia	Cao *et al*., 2024[5]; Tomic *et al*., 2022[2]
Cancer	Tomic *et al*., 2022[2]; Zheng *et al*., 2017[3]

Once these complications were defined, a directed literature search was conducted employing PubMed database to locate relevant studies assessing risk of complications associated with clinical markers linked to glycemia, weight and cardiovascular benefit in individuals with T2D. No year restriction was applied to review all relevant information available, but studies published from 2013 to the moment the search was launched (2024) were prioritised to obtain evidence with clinical relevance for the current T2D landscape. Previous studies were only assessed if no recent evidence was found about a topic. Regarding study designs, randomized clinical trials and observational studies (prospective and retrospective) were evaluated for inclusion, whereas meta-analysis were used as a source for additional studies of interest. Key search terms were divided into two categories: T2D-related complications [*myocardial infarction, stroke, heart failure, peripheral vascular disease, chronic kidney disease, diabetic neuropathy, retinopathy, dementia, cancer*], and clinical markers used to measure therapeutic objectives in T2D [*HbA1c, weight/body mass index (BMI), high-sensitivity C-reactive protein (hs-CRP)*]. The search strategy was defined as shown in the following table.

Target complication	Search strategy
Myocardial Infarction	"myocardial infarction"[All Fields] AND "type 2 diabetes"[All Fields] AND "risk"[All Fields] AND ("HbA1c"[All Fields] OR "weight"[All Fields] OR "body mass index"[All Fields] OR "hs-CRP"[All Fields])
Heart failure	"heart failure"[All Fields] AND "type 2 diabetes"[All Fields] AND "risk"[All Fields] AND ("HbA1c"[All Fields] OR "weight"[All Fields] OR "body mass index"[All Fields] OR "hs-CRP"[All Fields])
Stroke	"stroke"[All Fields] AND "type 2 diabetes"[All Fields] AND "risk"[All Fields] AND ("HbA1c"[All Fields] OR "weight"[All Fields] OR "body mass index"[All Fields] OR "hs-CRP"[All Fields])
Peripheral vascular disease	"peripheral vascular disease"[All Fields] AND "type 2 diabetes"[All Fields] AND "risk"[All Fields] AND ("HbA1c"[All

	Fields] OR "weight"[All Fields] OR "body mass index"[All Fields] OR "hs-CRP"[All Fields])
Chronic kidney disease	"chronic kidney disease"[All Fields] AND "type 2 diabetes"[All Fields] AND "risk"[All Fields] AND ("HbA1c"[All Fields] OR "weight"[All Fields] OR "body mass index"[All Fields] OR "hs-CRP"[All Fields])
Diabetic neuropathy	"diabetic neuropathy"[All Fields] AND "type 2 diabetes"[All Fields] AND "risk"[All Fields] AND ("HbA1c"[All Fields] OR "weight"[All Fields] OR "body mass index"[All Fields] OR "hs-CRP"[All Fields])
Retinopathy	"retinopathy"[All Fields] AND "type 2 diabetes"[All Fields] AND "risk"[All Fields] AND ("HbA1c"[All Fields] OR "weight"[All Fields] OR "body mass index"[All Fields] OR "hs-CRP"[All Fields])
Dementia	"dementia"[All Fields] AND "type 2 diabetes"[All Fields] AND "risk"[All Fields] AND ("HbA1c"[All Fields] OR "weight"[All Fields] OR "body mass index"[All Fields] OR "hs-CRP"[All Fields])
Cancer	"cancer"[All Fields] AND "type 2 diabetes"[All Fields] AND "risk"[All Fields] AND ("HbA1c"[All Fields] OR "weight"[All Fields] OR "body mass index"[All Fields] OR "hs-CRP"[All Fields])

HbA1c: glycated haemoglobin; hs-CRP: high-sensitive C-reactive protein.

The studies matching the search strategy were then filtered in a two-step optimized screening process: I) studies were assessed based on title and abstract directly on the database, and II) the selected studies were downloaded and a full text revision was performed. Exclusion criteria for each step is listed in the following table.

**Exclusion criteria for title & abstract screening**
Studies not assessing T2D patients or lacking a subanalysis for this population
Studies not reporting association of complications with HbA1c, weight/BMI, or hs-CRP
Studies assessing complications with cost quantification not feasible
**Exclusion criteria for full text screening**
Studies only reporting composite endpoints
Studies reporting no association between complications and HbA1c, weight/BMI, or hs-CRP
Studies reporting contradictory evidence for the same complication

BMI: body mass index; HbA1c: glycated haemoglobin; hs-CRP: high-sensitive C-reactive protein; T2D: type 2 diabetes.

In addition, specific searches were conducted to extract evidence on the rest of inputs required in the model (Tables S1 – S3). Whenever available, Spanish-specific evidence was collected for the following inputs: incidence of T2D complications; weight, HbA1c and hs-CRP distribution in the Spanish T2D population; T2D prevalence; proportion of T2D patient subgroups (treated, uncontrolled and/or with BMI ≥ 30 kg/m^2); and cost of complications.

Supplementary Tables

Table S2~~1~~. Sources of inputs included in the model.

Input	Source
Risk of weight-associated complications	
Myocardial Infarction	Bangalore *et al.*, 2018[6]
Heart failure	Aucott *et al.*, 2016[7]
Stroke	Bangalore *et al.*, 2018~~6~~[6]
Peripheral vascular disease	Aucott *et al.*, 2016[7]
Chronic kidney disease	Mohammedi *et al.*, 2018[8]
Diabetic neuropathy	Polemiti *et al.*, 2021[9]
Dementia	Chen *et al.*, 2023[10]
Cancer	Jonasson *et al.*, 2014[11]
Risk of HbA1c-associated complications	
Myocardial Infarction	Ceriello *et al.*, 2021[12]
Stroke	Ceriello *et al.*, 2021[12]
Peripheral vascular disease	Rozing *et al.*, 2019[13]
Chronic kidney disease	Penno *et al.*, 2013[14]
Retinopathy	Penno *et al.*, 2013[14]
Dementia	Ramírez *et al.*, 2015[15]
Risk of hs-CRP-associated complications	
Myocardial Infarction	Scirica *et al.* 2016[16]; Hwang *et al.* 2017[17]
Stroke	Scirica *et al.* 2016[16]; Hwang *et al.* 2017[17]
Incidence of complications	
Myocardial Infarction	Jodar *et al.*, 2020[18]
Heart failure	Jodar *et al.*, 2020[18]
Stroke	Jodar *et al.*, 2020[18]
Peripheral vascular disease	Sicras-Mainar *et al.*, 2022[19]
Chronic kidney disease	Sicras-Mainar *et al.*, 2022[19]
Diabetic neuropathy	Mundet *et al.*, 2008[20]
Retinopathy	Romero-Aroca *et al.*, 2017[21]
Dementia	Alsharif *et al.*, 2020[22]
Cancer	Global Cancer Observatory[23]
Weight distribution	European Health Survey in Spain, 2020[24]
HbA1c distribution	Mata-Cases *et al.*, 2016[25]<br>Díaz-Cerezo *et al.*, 2020[26]
hs-CRP distribution	Donate-Correa *et al.*, 2023
Interaction between weight and HbA1c	Gummeson *et al.*, 2017[27]
T2D prevalence	IDF, 2021[28]<br>BDCAP, 2017[29]
Proportion of treated T2D patients	Mata-Cases *et al.*, 2022[30]
Proportion of uncontrolled T2D patients	Vinagre *et al.*, 2012[31]
Proportion of uncontrolled T2D patients with BMI ≥ 30 kg/m^2	Romera *et al.*, 2020[32]
DALYs of complications	World Health Organization[33]; Ferrari *et al.*, 2024[34]; Eid *et al.*, 2023[35]
Costs of complications	RAE-CMBD[36]

BMI: body mass index; HbA1c: glycated haemoglobin; T2D: type 2 diabetes.

Table S3~~2~~. DALYs associated with complications.

Complication	DALYs	Source
Myocardial infarction	0.076	World Health Organization[33]
Heart failure	0.019	World Health Organization[33]
Stroke	0.041	World Health Organization[33]
Peripheral vascular disease	0.014	Eid *et al*., 2023[35]
Chronic kidney disease	0.104	Ferrari *et al*., 2024[34]
Retinopathy	0.133	World Health Organization[33]
Dementia	0.005	World Health Organization[33]
Diabetic neuropathy	0.069	World Health Organization[33]
Cancer	0.288	World Health Organization[33]

DALY: disability-adjusted life year

Table S43. Costs associated with complications based on DRGs (€, as of 2025).

Complication	DRGs[36]	Cost (€, as of 2025)
Myocardial infarction	190	3,997.60 €
Heart failure	194	3,761.94 €
*Stroke**	045/046/047	8,860.95 €
Peripheral vascular disease	197	3,992.30 €
Chronic kidney disease	470	3,428.49 €
*Retinopathy**	082	8,770.38 €
Dementia	042	4,986.95 €
*Diabetic neuropathy**	048	15,990.31 €
Cancer	041/110/136/240/281/284/ 346/281/284/346/381/382/ 461/500/530/690/691/694	4,927.49 €

*Cost per DRG (38th version, 2023) associated with the highest level of severity (level 4). DRG: diagnosis-related group

Table S54. Consensus-based checklist for cost-of-illness studies[37].

Item	Question	Answer*	Supportive information
Question/objective	1) Is a well-defined research question or objective stated?	Yes	Stated in the last paragraph of the Introduction section
Population	2) Is the study population described?	Yes	Described in the first paragraph of section 2.2 Modelling and statistical analysis (Methods)
Perspective	3) a) Is (are) the chosen study perspective(s) stated?	Yes	Stated in the last paragraph of the Introduction section
	b) If so, is (are) the chosen study perspective(s) justified?	Yes	The focus on the Spanish T2D population and NHS is made clear throughout the text
Epidemiological approach	4) Is the epidemiological approach reported (e.g., prevalence, incidence)?	Yes	Reported in Table S1 in Supplementary Materials
Costing approach	5) Is the costing approach reported (e.g., top-down, bottom-up)?	Yes	Reported in the third paragraph of section 2.2 Modelling and statistical analysis (Methods)
Data collection approach	6) Is the data collection process reported (e.g., prospective, retrospective)?	Yes	Described in the first paragraph of section 2.1 Literature search and data extraction (Methods)
Identification	7) a) Are all components of resource use identified that are relevant to the condition/disease, population, intervention, study objectives, and study perspective?	Partially/NA	This is not a cost-of-illness analysis, but rather an analysis evaluating the cost savings derived from avoiding complications, considering only the short-term impact. Therefore, it is not necessary to identify all resource use related to the disease, but only the overall cost of managing the corresponding complications at the hospital setting.
	b) If not, is a justification provided for excluding relevant components of resource use?	No	
Measurement	8) a) Are all included components of resource use measured?	NA	
	b) If not, is a justification provided for not measuring certain components of resource use?	NA	
Valuation	9) a) Are all included components of resource use valued in monetary terms?	Partially	DRGs are employed to identify general costs of hospitalization for the corresponding complications during the acute or initial phase - that is, costs of one hospitalization. These costs comprise the use of resources for the mentioned management.
	b) If not, is a justification provided for not valuing certain components of resource use?	NA	

Time horizon	10) a) Is the chosen time horizon specified?	Yes	Reported in the third paragraph of section 2.2 Modelling and statistical analysis (Methods)
	b) If so, is the chosen time horizon justified?	No	
Discounting	11) a) Are future costs discounted?	NA	
	b) If so, is a justification provided for the discount rate?	NA	
Sensitivity	12) a) Are all variables whose values are uncertain subjected to sensitivity analysis?	Yes	Reported in the fourth paragraph of section 2.2 Modelling and statistical analysis (Methods)
	b) If so, is a justification provided for which variables are subjected to sensitivity analysis?	Partially	Reported in the fourth paragraph of section 2.2 Modelling and statistical analysis (Methods)
	c) Are analyses done on relevant subgroups?	NA	
Cost sectors	13) Are the study results presented transparently by cost category/sector?	NA	
Generalizability	14) Do the authors discuss the generalizability of study results (e.g., comparing the results to other patient/client groups or/in other settings)?	Yes	Included in the Discussion section
Limitations	15) Do the authors discuss important limitations?	Yes	Discussed in section 4.1 Limitations (Discussion)
Ethical and distributional issues	16) a) Do the authors discuss ethical issues?	NA	
	b) Do the authors discuss distributional issues?	No	
Conflict of interest	17) Do the authors report any potential conflicts of interest?	Yes	Reported in Conflicts of Interest section

*Answer categories: Yes, No, Partially, Not Applicable (NA), and Unclear

Supplementary Figures

Figure S1. Percentage of reduction for each individual complication estimated for scenario I.

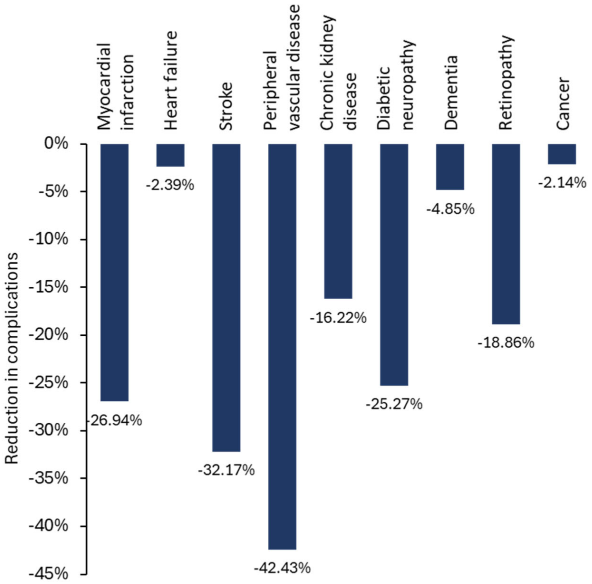

Figure S2. Percentage of reduction for each individual complication estimated for scenario II.

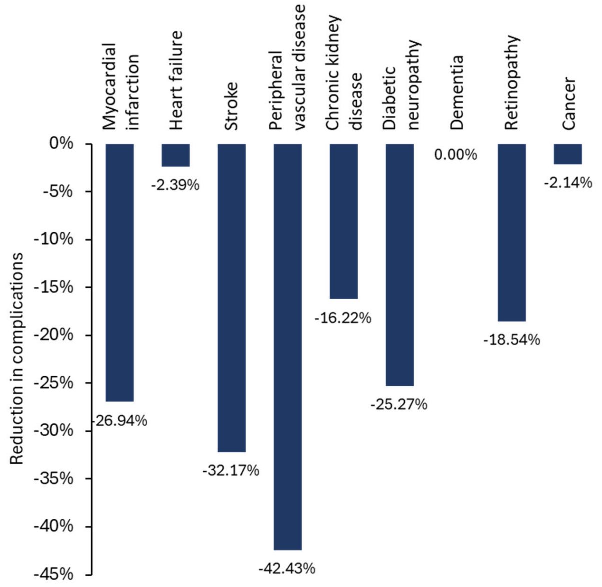

Figure S3. Percentage of reduction for each individual complication estimated for scenario III.

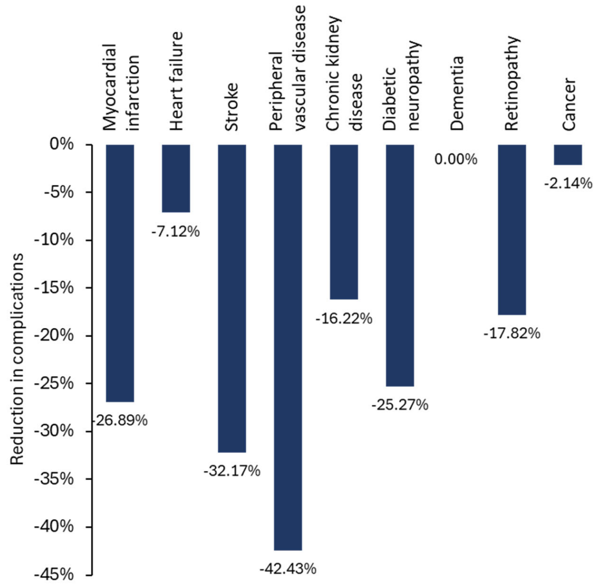

Figure S4. Patient distribution of the estimated number of avoided complications and their potential avoided DALYs and cost savings according to probabilistic sensitivity analyses. Patient distribution in number of complications, DALYs and costs intervals is depicted for scenarios I-III (A-C).

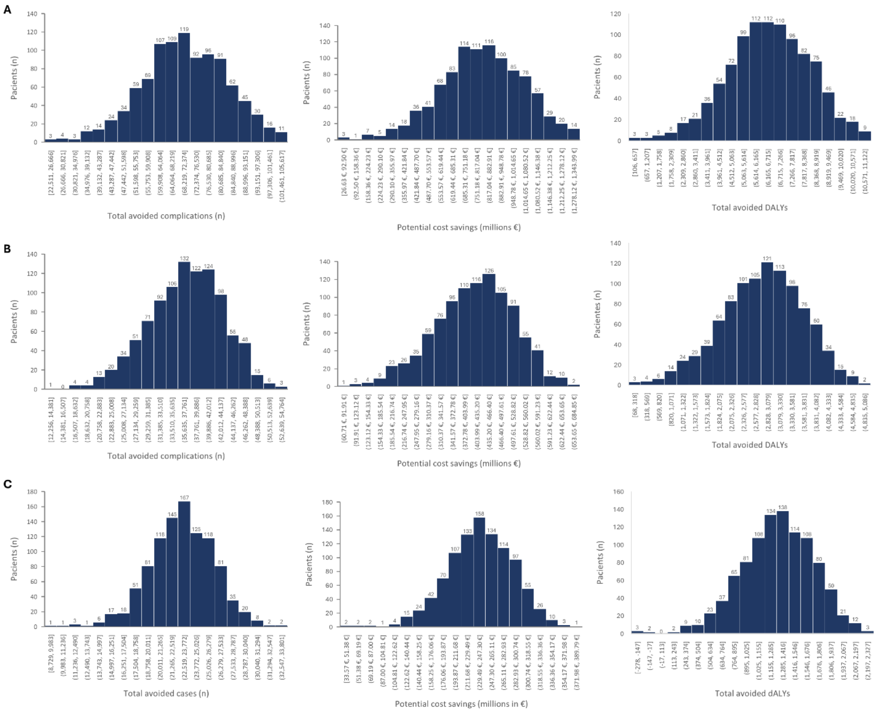

References

1. Shah, A., Isath, A. & Aronow, W. S. Cardiovascular complications of diabetes. *Expert Rev. Endocrinol. Metab.* **17**, 383–388 (2022).

2. Tomic, D., Shaw, J. E. & Magliano, D. J. The burden and risks of emerging complications of diabetes mellitus. *Nat. Rev. Endocrinol.* **18**, 525–539 (2022).

3. Zheng, Y., Ley, S. H. & Hu, F. B. Global aetiology and epidemiology of type 2 diabetes mellitus and its complications. *Nat. Rev. Endocrinol.* **14**, 88–98 (2018).

4. Lu, Y. *et al.* Vascular complications of diabetes: A narrative review. *Medicine* **102**, e35285 (2023).

5. Cao, F. *et al.* The relationship between diabetes and the dementia risk: a meta-analysis. *Diabetol. Metab. Syndr.* **16**, 101 (2024).

6. Bangalore, S., Fayyad, R., DeMicco, D. A., Colhoun, H. M. & Waters, D. D. Body Weight Variability and Cardiovascular Outcomes in Patients With Type 2 Diabetes Mellitus. *Circ. Cardiovasc. Qual. Outcomes* **11**, (2018).

7. Aucott, L. S. *et al.* Patterns of weight change after the diagnosis of type 2 diabetes in Scotland and their relationship with glycaemic control, mortality and cardiovascular outcomes: a retrospective cohort study. *BMJ Open* **6**, e010836 (2016).

8. Mohammedi, K. *et al.* Associations between body mass index and the risk of renal events in patients with type 2 diabetes. *Nutr. Diabetes* **8**, 7 (2018).

9. Polemiti, E. *et al.* BMI and BMI change following incident type 2 diabetes and risk of microvascular and macrovascular complications: the EPIC-Potsdam study. *Diabetologia* **64**, 814–825 (2021).

10. Chen, B., Sluiman, A. J., Khalid, W., Strachan, M. W. J. & Price, J. F. Risk of dementia associated with body mass index, changes in body weight and waist circumference in older people with type 2 diabetes: The Edinburgh Type 2 Diabetes Study. *Diabetic Medicine* **40**, (2023).

11. Miao Jonasson, J., Cederholm, J. & Gudbjornsdottir, S. Excess Body Weight and Cancer Risk in Patients with Type 2 Diabetes Who Were Registered in Swedish National Diabetes Register – Register-Based Cohort Study in Sweden. *PLoS One* **9**, e105868 (2014).

12. Ceriello, A. *et al.* HbA1c variability predicts cardiovascular complications in type 2 diabetes regardless of being at glycemic target. *Cardiovasc. Diabetol.* **21**, 13 (2022).

13. Rozing, M. P. *et al.* Changes in HbA1c during the first six years after the diagnosis of Type 2 diabetes mellitus predict long-term microvascular outcomes. *PLoS One* **14**, (2019).

14. Penno, G. *et al.* HbA1c Variability as an Independent Correlate of Nephropathy, but Not Retinopathy, in Patients With Type 2 Diabetes. *Diabetes Care* **36**, 2301–2310 (2013).

15. Ramirez, A. *et al.* Elevated HbA1c is Associated with Increased Risk of Incident Dementia in Primary Care Patients. *Journal of Alzheimer's Disease* **44**, 1203–1212 (2015).

16. Scirica, B. M. *et al.* Prognostic Implications of Biomarker Assessments in Patients With Type 2 Diabetes at High Cardiovascular Risk. *JAMA Cardiol.* **1**, 989 (2016).

17. Hwang, Y. *et al.* High-sensitivity C-reactive protein, low-density lipoprotein cholesterol and cardiovascular outcomes in patients with type 2 diabetes in the EXAMINE (Examination of Cardiovascular Outcomes with Alogliptin versus Standard of Care) trial. *Diabetes Obes. Metab.* **20**, 654–659 (2018).

18. Jodar, E. *et al.* Incidence and costs of cardiovascular events in Spanish patients with type 2 diabetes mellitus: a comparison with general population, 2015. *BMJ Open Diabetes Res. Care* **8**, e001130 (2020).

19. Sicras-Mainar, A. *et al.* Epidemiology and resource use in Spanish type 2 diabetes patients without previous cardiorenal disease: CaReMe Spain study summary. *Endocrinol. Diabetes Nutr.* **69**, 509–519 (2022).

20. Mundet, X. *et al.* Prevalence and incidence of chronic complications and mortality in a cohort of type 2 diabetic patients in Spain. *Prim. Care Diabetes* **2**, 135–140 (2008).

21. Romero-Aroca, P. *et al.* Differences in incidence of diabetic retinopathy between type 1 and 2 diabetes mellitus: a nine-year follow-up study. *Br. J. Ophthalmol.* **101**, 1346 (2017).

22. Alsharif, A. A. *et al.* Prevalence and Incidence of Dementia in People with Diabetes Mellitus. *J. Alzheimers Dis.* **75**, 607–615 (2020).

23. Global Cancer Observatory. https://gco.iarc.fr/en.

24. Instituto Nacional de Estadística. INEbase / Sociedad /Salud /Encuesta europea de salud en España / Resultados. https://www.ine.es/dyngs/INEbase/es/operacion.htm?c=Estadistica_C&cid=1254736176784&menu=resultados&idp=1254735573175 (2024).

25. Mata-Cases, M. *et al.* Direct medical costs attributable to type 2 diabetes mellitus: a population-based study in Catalonia, Spain. *European Journal of Health Economics* **17**, 1001–1010 (2016).

26. Díaz-Cerezo, S. *et al.* Resource use and costs in patients with poorly controlled type 2 diabetes mellitus and obesity in routine clinical practice in Spain. *Curr. Med. Res. Opin.* **36**, 1449–1456 (2020).

Formatted: Portuguese (Portugal)

27. Gummesson, A., Nyman, E., Knutsson, M. & Karpefors, M. Effect of weight reduction on glycated haemoglobin in weight loss trials in patients with type 2 diabetes. *Diabetes Obes. Metab.* **19**, 1295–1305 (2017).

28. International Diabetes Federation. Spain diabetes report 2000 — 2045. *Diabetes Atlas* https://diabetesatlas.org/data-by-location/country/spain/.

29. Ministerio de Sanidad - Sanidad en datos - Base de Datos Clínicos de Atención Primaria - BDCAP. https://www.sanidad.gob.es/estadEstudios/estadisticas/estadisticas/estMinisterio/SIAP/home.htm.

30. Mata-Cases, M. *et al.* Trends in the Degree of Control and Treatment of Cardiovascular Risk Factors in People With Type 2 Diabetes in a Primary Care Setting in Catalonia During 2007–2018. *Front. Endocrinol. (Lausanne).* **12**, 810757 (2022).

31. Vinagre, I. *et al.* Control of glycemia and cardiovascular risk factors in patients with type 2 diabetes in primary care in Catalonia (Spain). *Diabetes Care* **35**, 774–779 (2012).

32. Romera, I. *et al.* Clinical Inertia in Poorly Controlled Type 2 Diabetes Mellitus Patients with Obesity: An Observational Retrospective Study. *Diabetes Therapy* **11**, 437–451 (2020).

33. WHO. Department of Data and Analytics Division of Data, A. and D. for I. *WHO Methods and Data Sources for Global Burden of Disease Estimates 2000-2019. Global Health Estimates Technical Paper.* (2020).

34. Ferrari, A. J. *et al.* Global incidence, prevalence, years lived with disability (YLDs), disability-adjusted life-years (DALYs), and healthy life expectancy (HALE) for 371 diseases and injuries in 204 countries and territories and 811 subnational locations, 1990–2021: a systematic analysis for the Global Burden of Disease Study 2021. *The Lancet* **403**, 2133–2161 (2024).

35. Eid, M. A. *et al.* The global burden of peripheral artery disease. *J. Vasc. Surg.* **77**, 1119-1126.e1 (2023).

36. Ministerio de Sanidad. Secretaría General de Salud Digital, I. e I. del S. N. de S.-S. G. de I. S. *Norma Estatal Versión 38 de Los APR-GRD Del Registro de Altas de Los Hospitales Generales Del Sistema Nacional de Salud 2023*. https://www.sanidad.gob.es/estadEstudios/estadisticas/cmbd.htm (2025).

37. Schnitzler, L., Roberts, T. E., Jackson, L. J., Paulus, A. T. G. & Evers, S. M. A. A. A consensus-based checklist for the critical appraisal of cost-of-illness (COI) studies. *Int. J. Technol. Assess. Health Care* **39**, (2023).
